# Explainable Supervised Machine Learning Model To Predict Solvation Gibbs Energy

**DOI:** 10.1021/acs.jcim.3c00544

**Published:** 2023-08-21

**Authors:** José Ferraz-Caetano, Filipe Teixeira, M. Natália D. S. Cordeiro

**Affiliations:** † Department of Chemistry and Biochemistry − Faculty of Sciences, University of Porto - Rua do Campo Alegre, S/N, 4169-007 Porto, Portugal; ‡ Centre of Chemistry, University of Minho, Campus de Gualtar, 4710-057 Braga, Portugal

## Abstract

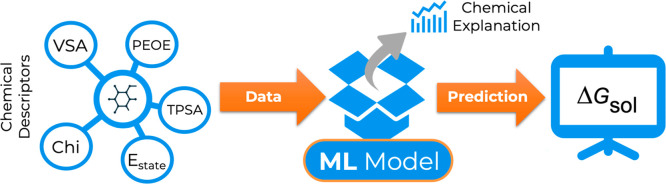

Many challenges persist in developing accurate computational models for predicting solvation free energy (Δ*G*_sol_). Despite recent developments in Machine Learning (ML) methodologies that outperformed traditional quantum mechanical models, several issues remain concerning explanatory insights for broad chemical predictions with an acceptable speed–accuracy trade-off. To overcome this, we present a novel supervised ML model to predict the Δ*G*_sol_ for an array of solvent–solute pairs. Using two different ensemble regressor algorithms, we made fast and accurate property predictions using open-source chemical features, encoding complex electronic, structural, and surface area descriptors for every solvent and solute. By integrating molecular properties and chemical interaction features, we have analyzed individual descriptor importance and optimized our model though explanatory information form feature groups. On aqueous and organic solvent databases, ML models revealed the predictive relevance of solutes with increasing polar surface area and decreasing polarizability, yielding better results than state-of-the-art benchmark Neural Network methods (without complex quantum mechanical or molecular dynamic simulations). Both algorithms successfully outperformed previous Δ*G*_sol_ predictions methods, with a maximum absolute error of 0.22 ± 0.02 kcal mol^–1^, further validated in an external benchmark database and with solvent hold-out tests. With these explanatory and statistical insights, they allow a thoughtful application of this method for predicting other thermodynamic properties, stressing the relevance of ML modeling for further complex computational chemistry problems.

## 1 Introduction

Predicting Gibbs free energy of solvation (Δ*G*_sol_) has always been challenging. This thermodynamic property has rendered many in silico computational methods for diverse and complex hydration mechanisms.^1−5^ Given its well-known role in chemical processes, this property is key for describing drug delivery systems,^6,7^ sustainable synthesis,^8^ and electrochemical performance.^9,10^ But despite recent breakthroughs, many challenges persist in creating models with acceptable accuracy.^11^ In particular, the design of comprehensive models capable of handling several hydration mechanisms of different solute–solvent pairs,^12,13^ beyond predictions for selected chemical groups.

Computational chemists often lack sufficient experimental data to approach increasing complex systems.^13−15^ To address this, various strategies to predict solvation free energy relied heavily on quantum mechanics (QM)^16−18^ or molecular dynamics,^19,20^ while some strategies used classical mechanics to describe hydration complexes with the Boltzmann equation, governing solvents’ behavior in an isotropic medium.^21−23^ This continuum solvation model, while it performs well at an acceptable computational cost, only considers small-molecule systems at a quantum level,^24^ statistically underperforming on complex examples. Also, molecular dynamics-based models yield considerable deviations for Δ*G*_sol_ predictions, mostly around the 4 kcal mol^–1^ mark.^3,25^ Another traditional strategy used atomistic characterization of the solvation shell through chemical hydration mechanisms,^26,27^ despite costly molecular simulations. Requiring molecular and force field dynamic calculations of each single structure,^28,29^ this approach only works for a handful of noncomplex simulations. While accurate, they present a time-consuming process by computing each new molecule parametrizations, adequate solute–solvent interactions, or entropic effects.

As both traditional approaches are sustained on extensive mathematical representations of chemical mechanisms, popular alternatives consider relinquishing this framework for a more quantitative approach. Quantitative structure–activity relationships (QSAR) based on Data Science methods are used to predict the outcome of a target property with rapid and extensive screening of a wide range of experimental data sets.^30−33^ This straightforward strategy is quite popular as scientists sometimes only want to predict a specific property, rather than characterizing all interactions within the chemical space. Using statistical regression analysis, Machine Learning (ML) methods have successfully predicted various thermodynamic properties, providing a computational depiction of the desired problem with substantial speed and accuracy.^34−40^ By representing each molecular entity through an array of quantitative descriptors, they work by translating them into key chemical features for property determination.^41,42^ Although ML strategies sometimes fail to make predictions under the 1 kcal mol^–1^ accuracy threshold,^43^ Δ*G*_sol_ calculation models have already revealed Mean Absolute Error (MAE) prediction values well below this barrier. A recent study^11^ has lowered this mark to 0.5 kcal mol^–1^ for Δ*G*_sol_ of organic compounds, as models for specific types of molecular families can improve prediction results.

Several frameworks have reported ML solvation models for accurate prediction of hydration free energy for a wide array of different solvent–solute complexes.^13,15,19,33,44^ Successful ML studies notably use neural network algorithms for Δ*G*_sol_ prediction. Using the group-contribution method for calculation of pairwise atomic interactions, Lim et al. report an artificial neural-network (ANN) model built on a base set of 6239 experimental measures of both aqueous and nonaqueous solvents.^5^ From these atomic feature vectors, the data undergo a pretraining process of atomistic representations of different elements distinguished by Morgan identifiers, handling complex atom connectivity. This encoding process analyzes several million molecular structures to extract key features correlated to free energy, feeding the ANN to predict Δ*G*_sol_ for the selected input database. Their overall MAE prediction yielded 0.19 kcal mol^–1^ and 0.76 kcal mol^–1^ for hydration benchmarking data sets, as this ANN strategy depicts one of the most powerful ML models for Δ*G*_sol_ prediction. Other flagship ML methods used graphical neural networks (GNN),^33^ delivering computational molecular descriptions based on graphs with nodes representing atoms and edges as bonds. These models require a considerable amount of input data to perform accurate predictions. As Δ*G*_sol_ experimental results are not usually in a significant number, models often use QM modeling to calculate solvation free energies and use this data to train the subsequent model. Despite prediction errors being below 0.4 kcal mol^–1^, these calculations amass a significant computational cost and do not take into account key parameters that would contribute to a different experimental result (with different solvent–solute interactions). Given this experimental bias, Low et al. tackled the issue by exclusively encoding chemically intuitive solvation features, namely partial atomic charges and solvent dielectric constants.^33^ Discarding the need for complex QM calculations, these GNN combined intuitive features with graph representation, computing Δ*G*_sol_ MAE prediction values of 0.4 kcal mol^–1^ for other organic solvents.

Additional methods based on ML strategies for Δ*G*_sol_ hydration free energy prediction have also been reported with particular differences in algorithm framework, from learning-to-rank to featurization methods.^32,45,46^ Aside from ANN and GNN models, only methods using transfer learning strategies^47^ or deep neural netowrks^48^ have achieved MAE prediction errors below 0.5 kcal mol^–1^. However, their increase in accuracy often comes with a trade-off on model representativity, as good predictions are only achieved when testing a small unrepresentative sample of the initial data set. These neural network models also present three key liabilities. First, strategies diverge on the type of input data used to make predictions, as chemical descriptors based on experimental data do not necessary depict all features relevant to predict a certain property. As each model uses different features, they are not transferable and underperform when they are considered for another ML-based method with a different testing set.^49^ Second, data availability jeopardizes model development, as great detail in experimental data for Δ*G*_sol_ limits models to specific free energy determinations, relinquishing important arrays of aqueous or organic solvents. Finally, most of these models lack explanatory arguments for their statistical predictions, enhancing the “black-box” mantra of ML-based predictions.^50,51^ With the exception of Low et al.^33^ (while still using QM calculations), previous models fail to describe the physical meaning behind each prediction.^52^

This work aims precisely to tackle these three issues. We present a supervised ML model to predict Δ*G*_sol_ from a wide array of experimental results. Our starting database amasses numerous documented experimental values, completed with open-source molecular physiochemical descriptors, tackling the transferability of method design. By using a diverse experimental data set of various solvent–solute pairings, we were able to achieve comparable accuracy scores with neural network models for organic and aqueous solvents, without additional QM determinations. Unlike previous complex neural-network models, we use two simple regressor algorithms: Random Forest (RF) and Gradient Boosting (GB). Both use an ensemble series of independent trees, as RF works with recursive partitioning and GB’s trees correct residual values in each prediction. Our determinations were able to capture the physical meaning behind each prediction, as the quantification of chemical feature importance allowed us to name important descriptors to explain Δ*G*_sol_ calculation. Having these accurate Δ*G*_sol_ values can be useful in predicting other chemical properties such as enthalpies, molecular permeability, or TOX21 classifications (as denoted in previous studies^53−55^). We thus present a simple, explainable, and wide scope model for Δ*G*_sol_ predictions, with the potential to be expanded to other open-source experimental databases.

This Article starts by showcasing our model building framework, emphasizing selected ML algorithms and chemical descriptor selection. After unfolding the experimental starting database, we present our validation studies for Δ*G*_sol_ prediction, including a cross-validation with an external benchmark database used to accredit previous ML models. We then analyze the explanatory power of our model, studying the physical significance of relevant chemical descriptors. By withholding several organic solvents, we further test model performance on predicting Δ*G*_sol_ across a second validation trial run.

## 2 Methodology

### 2.1 Database and Descriptor Generation

The database for the ML model contains a variety of solvent–solute pairs with known experimental Δ*G*_sol_ values. Data entries were collected from two separate databases. The FreeSolv^56^ library, with 642 experimental aqueous Δ*G*_sol_ determinations, and the Solv@TUM^57^ database, with 5597 entries for nonaqueous solvents. Both databases were chosen, given their wide scale of solute–solvent pairs, amassing 6239 experimental values across light- and heavy-atom solutes with a diverse solvent structure and with small value uncertainties.

Experimental Δ*G*_sol_ values range from −14 to 4 kcal mol^–1^, and each solute–solvent pair is represented by their chemical formula, IUPAC name, SMILES string, and InChlKey. The chemical descriptors used in the model were generated for every solvent and solute using RDKit^58^ software, version 2022.09.4, running on top of Python 3.9.^59^ The database and the 213 calculated descriptors are presented in the Supporting Information (SI) and are available at the zenodo repository.^60^

### 2.2 Machine Learning Model Framework

**Figure 1:**
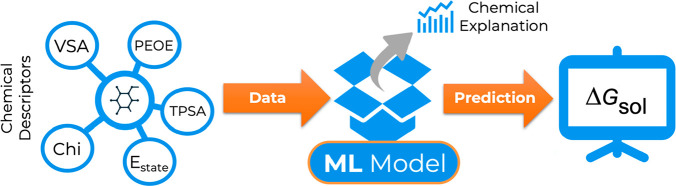
Workflow for Δ*G*_sol_ prediction using an ML-based model.

In Figure 1 we depict the workflow of our model. On our initial database, each solvent–solute pair is represented by 213 molecular descriptors. Descriptors encoding significant information are used to present physicochemical characteristics of compounds to build the relationship between structure and Δ*G*_sol_. Through regression algorithms, ML models will be able to make predictions based on the information encoded in each chemical feature. After calculation of RDKit descriptors, the database was divided in three different subsets. Following the methodology of a successful ML prediction model,^33^ we split the data in an 80:10:10 ratio for training, testing, and validation (respectively). These sets were then used to build, train, and statistically validate the model through an ML algorithm. The ML model is exposed to the training set, learning the key relationships to yield predictions, and then makes predictions for the unseen testing set (as we record prediction accuracy). Using a systematic grid search for the optimum algorithm hyperparameters, we performed a 10-fold cross-validation scheme on the ML model. Prediction accuracy reports the average over 10 different runs with the same random seed. A sample of the code and each model optimization with the best hyperparameter determination is presented in the SI and at https://github.com/jfcaetano/GibbsML.

After model optimization, data from the testing set were used to predict Δ*G*_sol_ values, as performance was assessed through several parameters: (i) coefficient of determination (Score), (ii) maximum absolute error (MAE), (iii) root mean squared error (RMSE), and (iv) standard deviation prediction of the cross-validation calculations (SPD). Using this statistical information and descriptor importance, we then described model performance according to the explanatory features extrapolated from our predictions. To further compare our models with other benchmark ML studies, we conducted two validation tests. First, we used our optimized model to predict Δ*G*_sol_ values from the FreeSolv^56^ database and compared its performance with other studies using the same database. Second, to assess the generalizability of our ML model to unseen solvents in the starting database, we conducted a holdout test on nine different solvents. This was done by removing the selected solvents from the initial data set, prior to the training and validation steps, while comparing our results with previous models.

## 3 Results and Discussion

### 3.1 Model Performance

**Figure 2:**
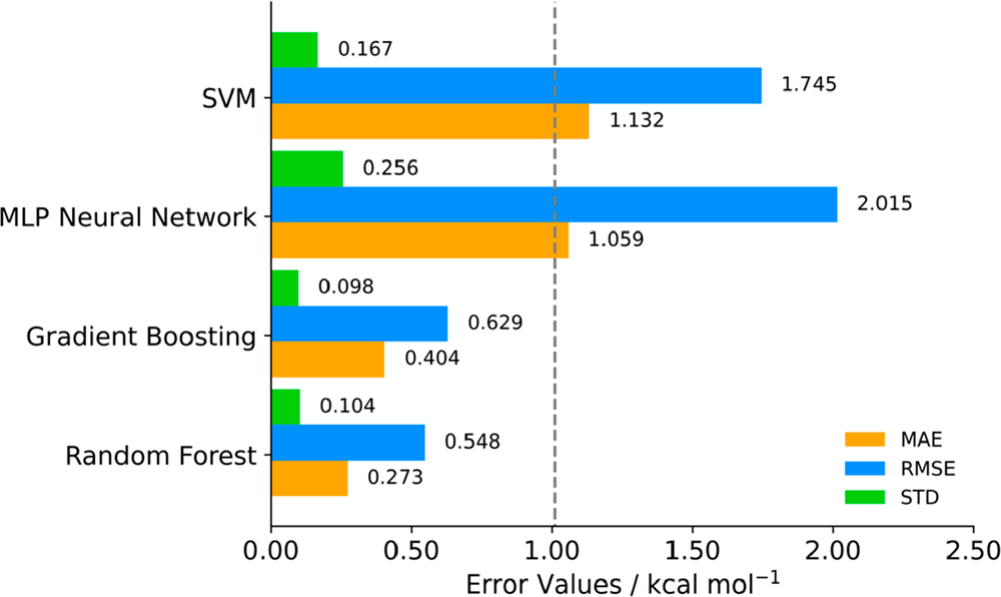
Error parameters pretest for different unoptimized ML regressor algorithms for Δ*G*_sol_ prediction dataset using a train:test:validation split of 80:10:10. Gray band represents a 1 kcal mol^–1^ threshold.

Before selecting the preferred algorithm to use in our ML model, we carried out a preselection test. We tested four different ML regressor algorithms on the basis of their computational cost and previous successful attempts on predicting other thermodynamic properties: Random Forest (RF), Gradient Boosting (GB), Support Vector Machines (SVM), and Multi-Layer Perceptron Neural Network (NN). We verified model results with a random 80:10:10 test:train:validation split, recording the MAE, RMSE, and STD for Δ*G*_sol_ prediction. Figure 2 shows the performance of each algorithm.

Comparing each algorithm’s performance with the 1 kcal mol^–1^ error benchmark,^43^ both RF and GB have all statistical indicators below this threshold. NN and SVM also present interesting results but are clearly outperformed by the ensemble algorithms. Given the structure of our database with 213 descriptors for each entry, perhaps NN methods might exceed these results if each solute–solvent pair had more than 213 features. Hence, that is why ensemble methods are better suited for databases with hundreds of descriptors.

**Figure 3:**
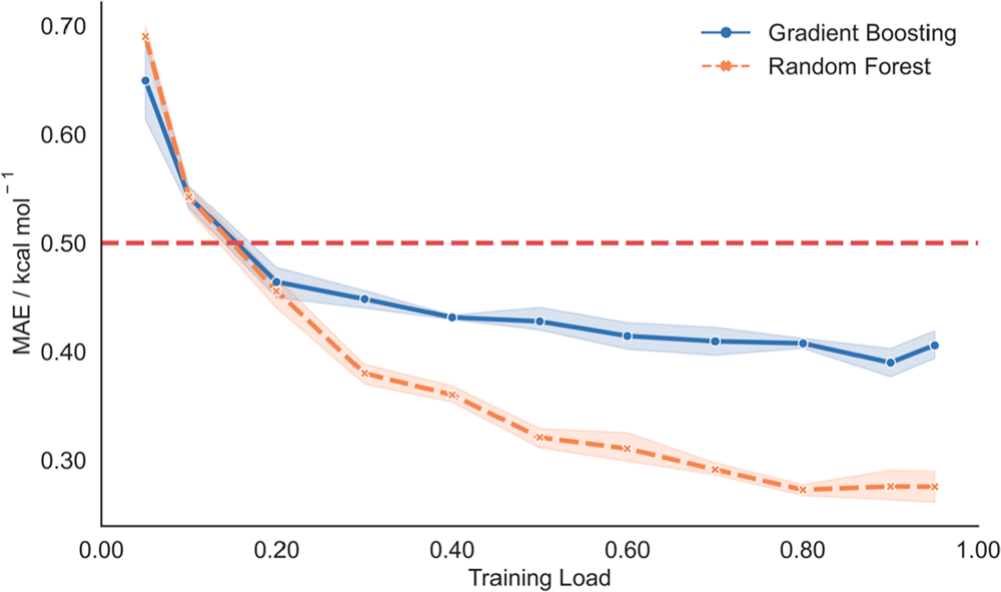
Fitness prediction plot for Δ*G*_sol_ MAE prediction using the ML-based model with RF and GB algorithms. Red band represents a 0.5 kcal mol^–1^ threshold.

Prior to initial Δ*G*_sol_ prediction with comparable train:test:validation sets, we evaluated model performance with increasing training loads, using data from all 213 chemical descriptors. This was done to secure model representability throughout the entire database. Figure 3 depicts the evolution of MAE prediction values for RF and GB algorithms using different train:test ratios.

**Figure 4:**
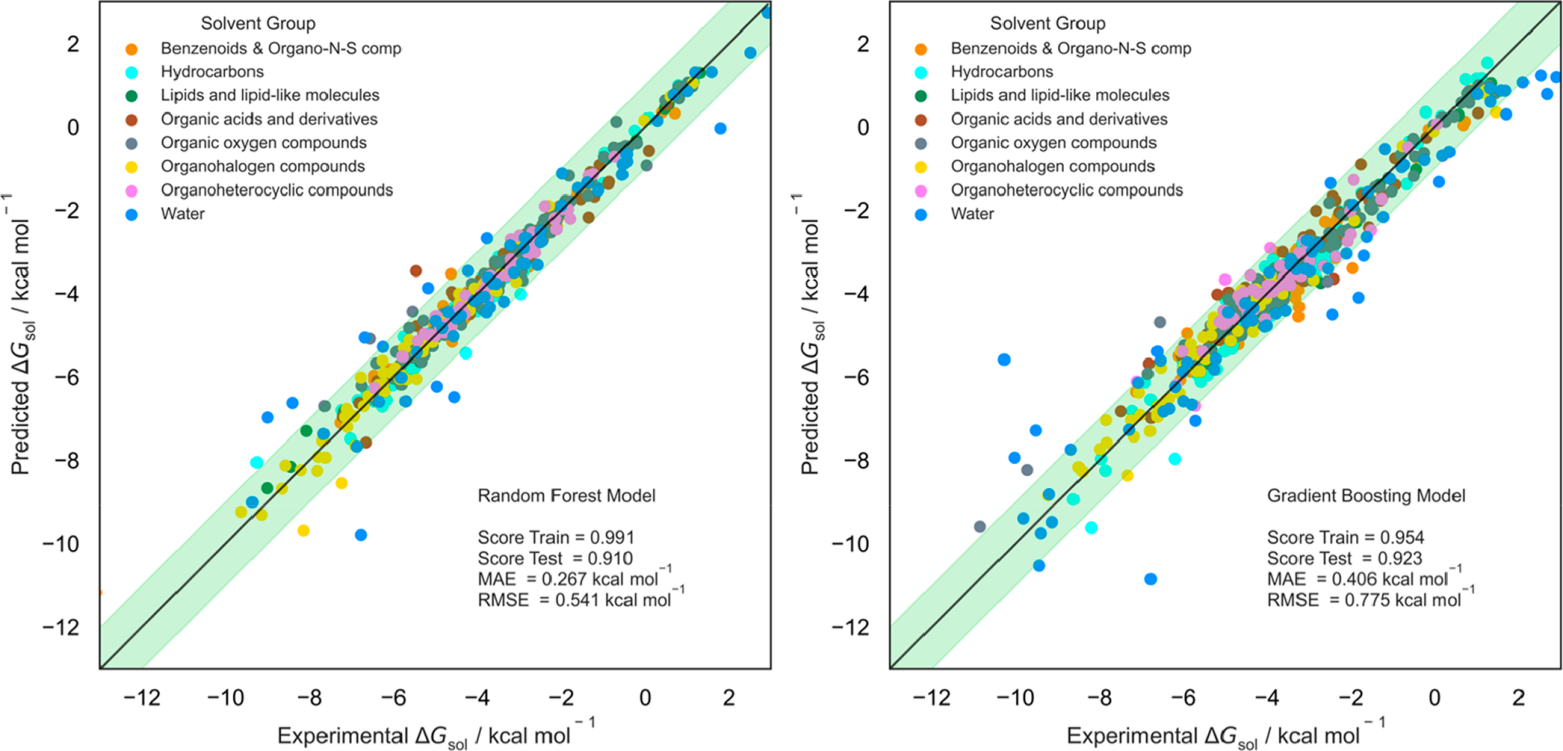
Fitness prediction plot for Δ*G*_sol_ prediction using a ML model with RF (left) and GB (right) algorithms. Green band is within the 1 kcal mol^–1^ threshold.

With increasing training load, the MAE for free energy prediction on both algorithms decreases. From a 20% training load, they can make predictions with MAEs below 0.5 kcal mol^–1^, matching previously cited ML models. Given the establishment of a stable state from 50% onward, considering the trade-off between accuracy and representability, we confirmed the suitability of this model for Δ*G*_sol_ prediction. We then calculated the statistical performance of RF and GB models with all 213 calculated descriptors, using the 80:10:10 train:test:validation sets, as presented in Table 1 and in Figure 4.

**Table 1:** Results for Model Performance of Δ*G*_sol_ Prediction for Preoptimized RF and GB Algorithms

Regressor Algorithm	Score Train	Score Test	MAE/kcal mol^–1^	RMSE/kcal mol^–1^	SDP/kcal mol^–1^
Random Forest	0.94 ± 0.01	0.991 ± 0.001	0.28 ± 0.03	0.59 ± 0.08	0.106 ± 0.007
Gradient Boosting	0.922 ± 0.004	0.952 ± 0.002	0.40 ± 0.02	0.63 ± 0.02	0.10 ± 0.03

Both algorithms present favorable results, with MAE scores well below the 1 kcal mol^–1^ threshold. Compared with other ML models, Low et al. neural network model^33^ yielded a similar MAE, below 0.5 kcal mol^–1^. Without using a powerful algorithm or a densely populated database, our results show that both ensemble regression algorithms present a competitive model for Δ*G*_sol_ prediction. They have certainly benefited from a diverse set of solvent–solute pairs on the testing set, as most predictions average a standard error of 0.10 kcal mol^–1^. Both algorithms present a residual difference in the calculated parameters, stressing the statistical significance of the model given its database architecture and compound diversity. With these results, we proceeded with model prediction optimization starting by analyzing descriptors significance in Δ*G*_sol_ calculation.

### 3.2 Model Descriptor Performance

**Figure 5:**
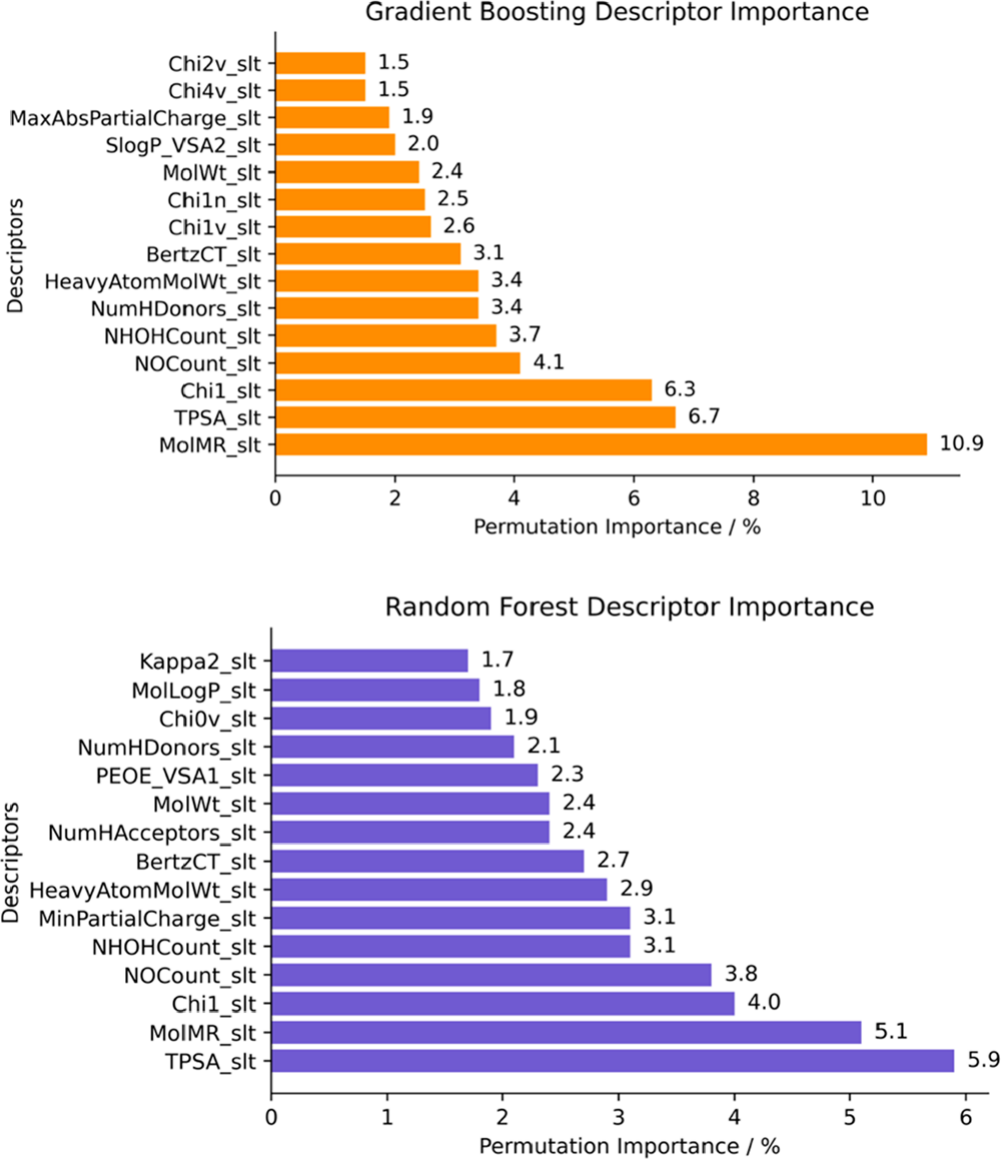
Feature permutation importance for most relevant descriptors in GB (orange) and RF (violet) model predictions.

To improve our models, we determined the prevalent chemical feature importance in Δ*G*_sol_ prediction. We used permutation importance (PI) to provide a description of feature performance, revealing a quantitative relationship with the predicted target through different permutations. Both RF and GB algorithms have roughly over 35% of all 213 features with 0 or less permutation importance, while the top 20 amass 50% of PI. Figure 5 displays the most important descriptors for Δ*G*_sol_ predictions, while full descriptor permutation importance is presented in the SI. For these algorithms, the solute–solvent feature ratio is roughly 80:20 as solute descriptors have the most preponderance. This gives a pivotal role to the solute descriptors as the most statistically influential in Δ*G*_sol_ prediction. Considering the top 15 most preponderant descriptors (Figure 5), the GB algorithm favors various chi connectivity indices (χ); surface area features such as TPSA (topological polar surface area), and VSA state (volume surface area); structural descriptors, such as molecular weight; and electronic such as molar refractivity (MolMR) and electrotopological state atom (E-state). As for the RF model, we can identify almost the same top 15 descriptors, aside from a more even PI distribution and including other features.

Since Δ*G*_sol_ represents a free energy variation due to a phase change to the solvated state, it would be expected that molecular weight could be the most relevant feature, as larger molecules are more unstable in their gas phases, regardless of the solvent–solute interactions. But with the GB model, the key molecular descriptors reveal a preeminent electronic feature. Molar Refractivity (the total polarizability of a mole of a substance) accounts for atom and electronic interactions within a molecular environment. Among other high-ranked descriptors are surface area features, as TPSA gives the overall topological polar surface area of the molecule and VSA features represent the electrotopological state (E-state) within a van der Waals surface area. The χ descriptors are structural attributes represented by quantitative molecular connectivity with molecular fragment structural information. The various subtypes of each χ descriptor represent increasing levels of structural information, encoding simple (single, linear, etc.) to complex structures (clusters, rings, etc.).

**Figure 6:**
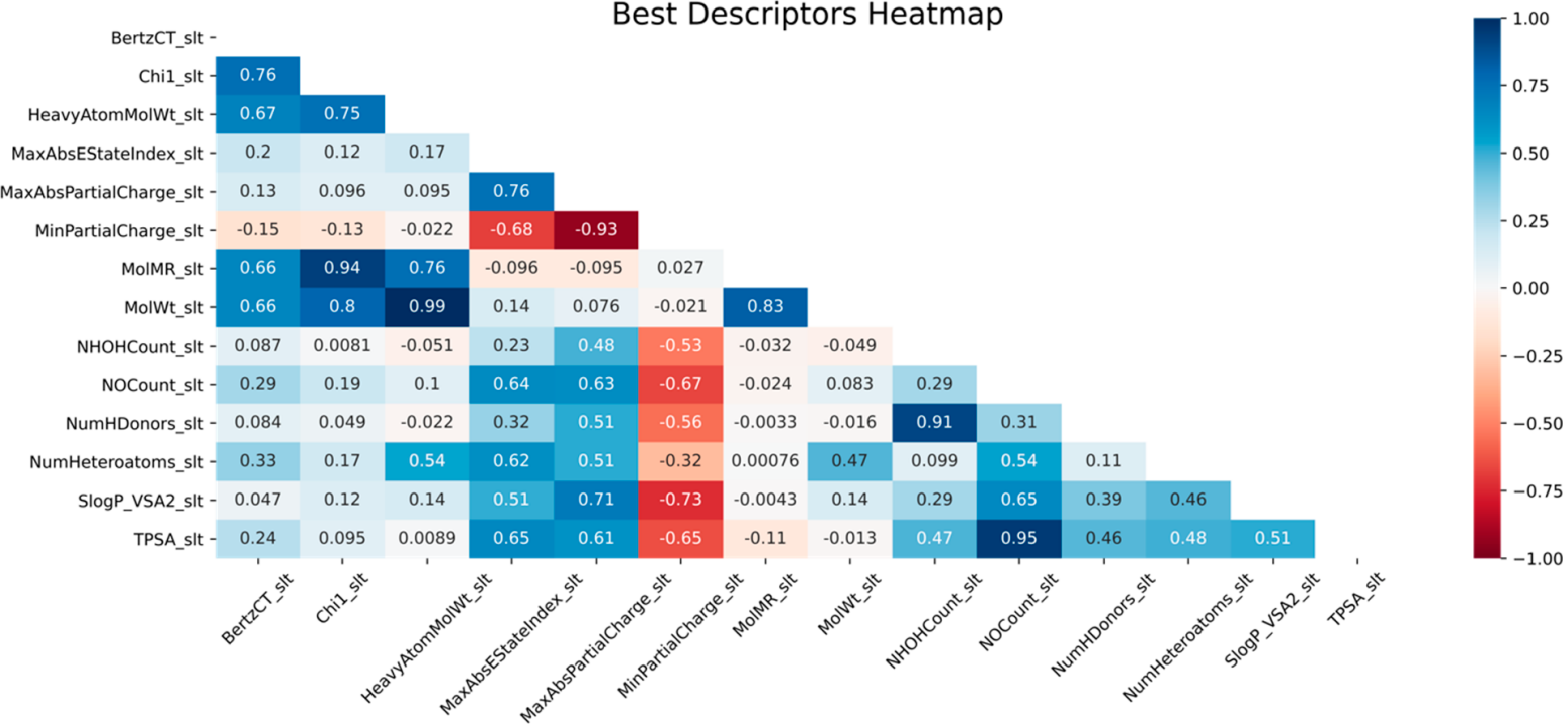
Correlation heatmap for top key descriptors in ML model prediction with the GB algorithm.

Given the diversity of descriptor type PI distribution, a straightforward selective contribution of a descriptor group cannot be proclaimed. To assess this, we evaluated descriptor importance with feature heatmap correlation. Figure 6 presents the correlation heatmap for the 14 descriptors with higher permutation importance, giving a numerical description of the behavior between two specific descriptors (while removing descriptors from the same family to avoid overcorrelation figures, e.g., χ-based features). The performance of high ranked RF and GB descriptors is roughly the same (more detailed charts are presented in the SI).

**Figure 7:**
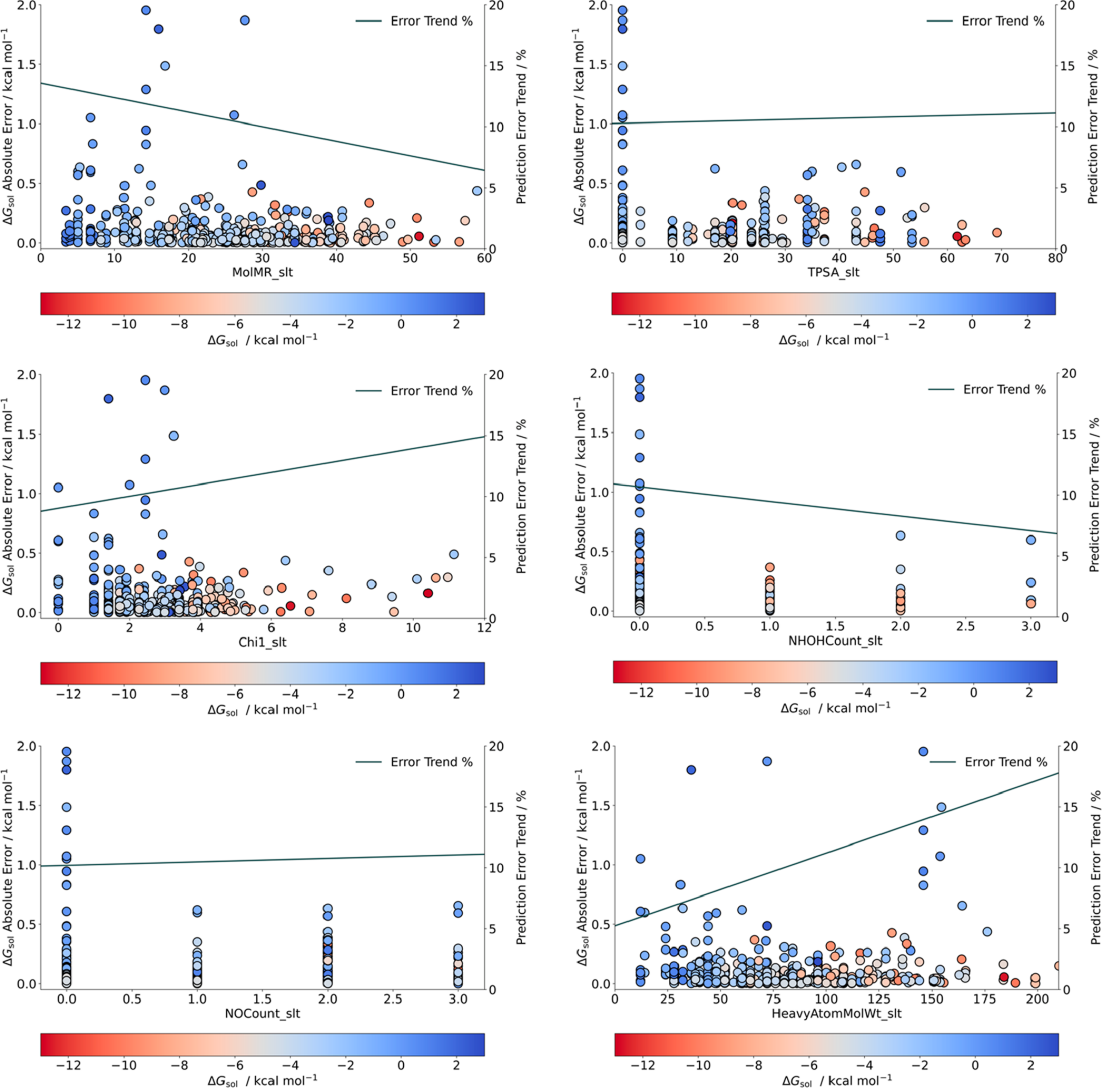
Feature dependency on Δ*G*_sol_ prediction for relevant descriptors with the RF algorithm.

While most prominent descriptors are not positively correlated with each other, there is a notable exception concerning electronic (MolMR) and structural features (1χ, MolWt, and HeavyAtomCounts). Although this correlation might be understandable by similarities in how these quantitative descriptors are generated, this behavior is not only a statistical trend. If we remove the two features concerning Partial Charge, all descriptors have a neutral or positive correlation. While there is room for descriptor overfitting with highly corelated features, these results show that descriptor differentiation may grasp important chemical information without overinfluencing the statistical result. Their mathematical performance affects each of the model’s predictions, arguing if all features were highly correlated, little to no explanatory information could be made. This, however, can only be proven by analyzing how prediction errors are influenced by each descriptor’s response. If a trend can be established among strong descriptor variation, an explainable argument can be outlined for how model predictions are made. Therefore, we plotted the absolute error prediction variation and error trend with key descriptor performance, comparing with the experimental Δ*G*_sol_ value in Figure 7.

**Figure 8:**
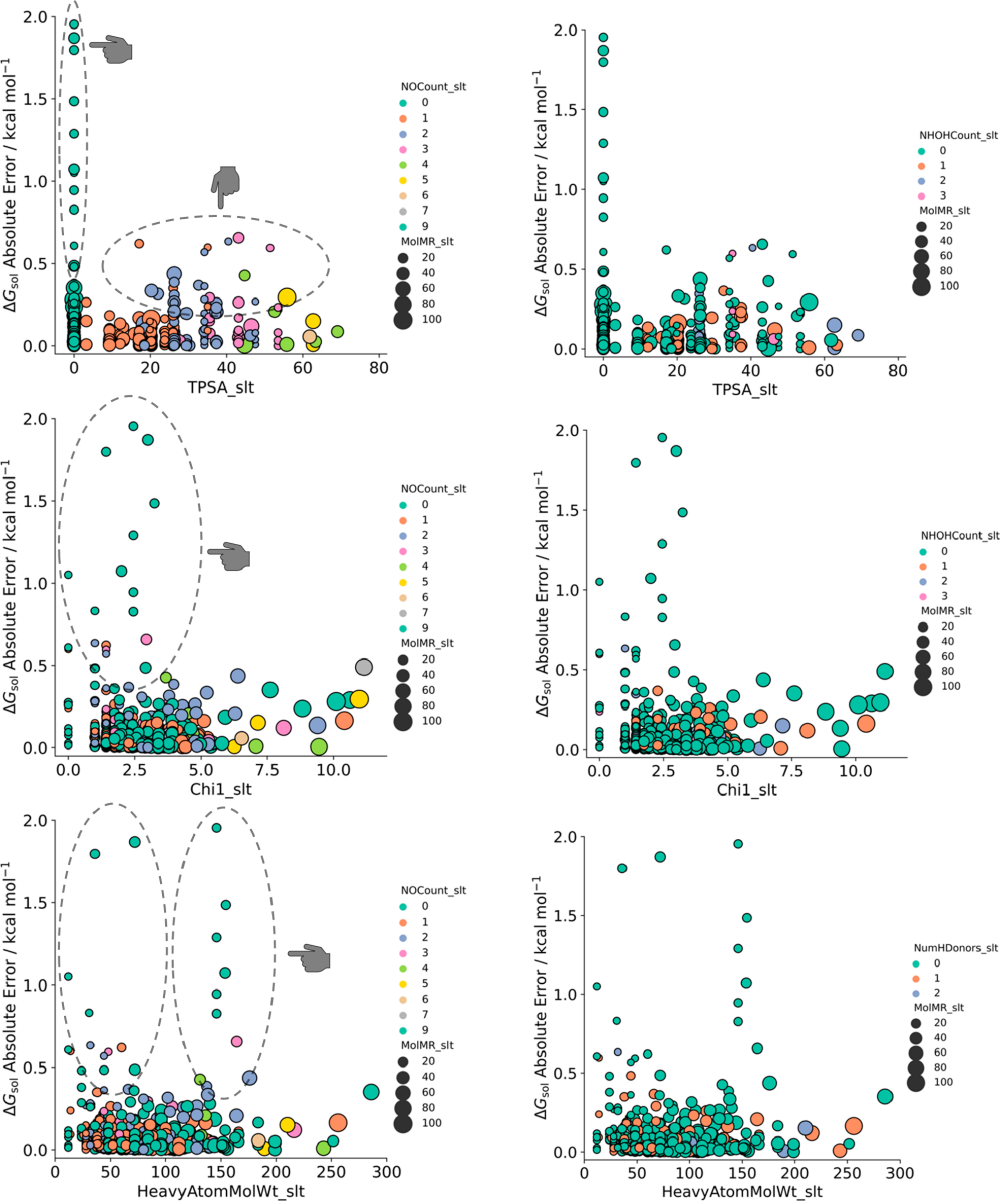
Δ*G*_sol_ prediction error distribution across relevant descriptors with feature cross-referencing.

Figure 7 presents representative descriptor prediction errors across key feature groups in the RF algorithm prediction: MolMR (electronic), TPSA (surface area) and 1χ, NHOHCount, NOCount, and HeavyAtomMolWt (structural). These descriptors combined outline approximately 40% of permutation importance (adding the amassed correlation of χ descriptors). Three different trends can be identified. First, low MolMR values are linked to higher prediction errors, as they tend to decrease as molar refractivity increases. It indicates that molecules with low polarizability (a low electric dipole moment in proportion to an electric field) with Δ*G*_sol_ over −2 kcal mol^–1^ have a tendency of low prediction accuracy in our model. The dispersion seen in the chart illustrates that lower MolMR data points are not clustered in lower error values, as it is clear as the value increases. While it is an error trend lower than 15%, it is noteworthy how it differentiates from the second trend we highlight. In TPSA error distribution, while low values have high error predictions, the data points are more dispersed as the surface area increases. This leads to a trend in prediction error for molecules with low Δ*G*_sol_. High polar surface area can amass different molecular characteristics, which makes it harder to identify a behavior pattern that eases prediction. As absolute error percentage remains stable, and the contrast with polarizability is interesting: a large molecule with greater surface area yielding an inaccurate prediction can be attenuated, as bigger molecules are more polarizable. This antagonic relationship shows how balancing descriptor influence is essential due to important contributions from cross-referencing features from different families. The third and final trend concerns structural descriptors. 1χ and HeavyAtomMolWt have an increasing slope as the error trend moves across higher descriptor values. It confirms our previous conclusion that bigger molecules have a tendency for high error predictions, especially in the Δ*G*_sol_ −5 to 3 kcal mol^–1^ range. As for the number of NHOH and NO groups, given its negligible trend when comparing with important electronic and surface area descriptors, we argue that there is not a forthright dominant correlation with molecule complexity and the variation of Δ*G*_sol_ prediction errors. With this information, it is essential to have a holistic view on how these preeminent descriptor groups influence Δ*G*_sol_ predictions. Figure 8 plots the correlation among several key descriptors, grouped across their prediction error percentage.

While there is not a major solvent group responsible for higher prediction errors, as seen in Figure 4, it is possible to describe some particular descriptor behaviors. The error distribution allows a detailed description of what types of molecules are more difficult to predict. Starting with the MolMR, high prediction deviation stems from values up to 50, verifiable across different distributions from other features in Figure 7. In the case for TPSA, there is a low error cluster located when there is no surface area (TPSA = 0 Å), and then error predictions spread out when values are over 10. This second cluster is majority populated with molecules with at least one NO group, an absence of NHOH groups, and no H donors. A mildly complex molecular structure is argued as we compare with results for structural descriptors such as χ and Molecular Weight: a short-range between 2 and 4 for 1χ, indicating a particular fragment structure with low complexity, while displaying a weight diversity up to 200 g mol^–1^. Comparing key electronic and structural features, free energy distribution displays the same pattern when comparing overall error and χ performance. This connectivity index suggests that small structural differences can yield high prediction errors, as the model struggles in the Δ*G*_sol_ −5 to 3 kcal mol^–1^ interval. However, the MolMR distribution narrows these cases (up to MolMR < 50), which presumes that our model learned chemical features behind these predictions (e.g., structure differences in isomers and diastereomers). Despite different distributions of TPSA, VSA, and correlation with previous features, the data are assembled roughly the same way, indicating a consistency of this pattern across relevant descriptors. Considering the solvent group distribution presented in Figure 4, there is a noteworthy prediction bias observed when the solvent is water for low Δ*G*_sol_ values. Therefore, a representative error depiction in solvation free energy occurs in solvent–solute pairs where there is an aqueous solvent and mildly complex solute structure, with increasing polar surface area and decreasing polarizability. In opposition, there is a significant performance improvement for organic solvents with wide polar surface areas and increasing dipole movement.

**Figure 9:**
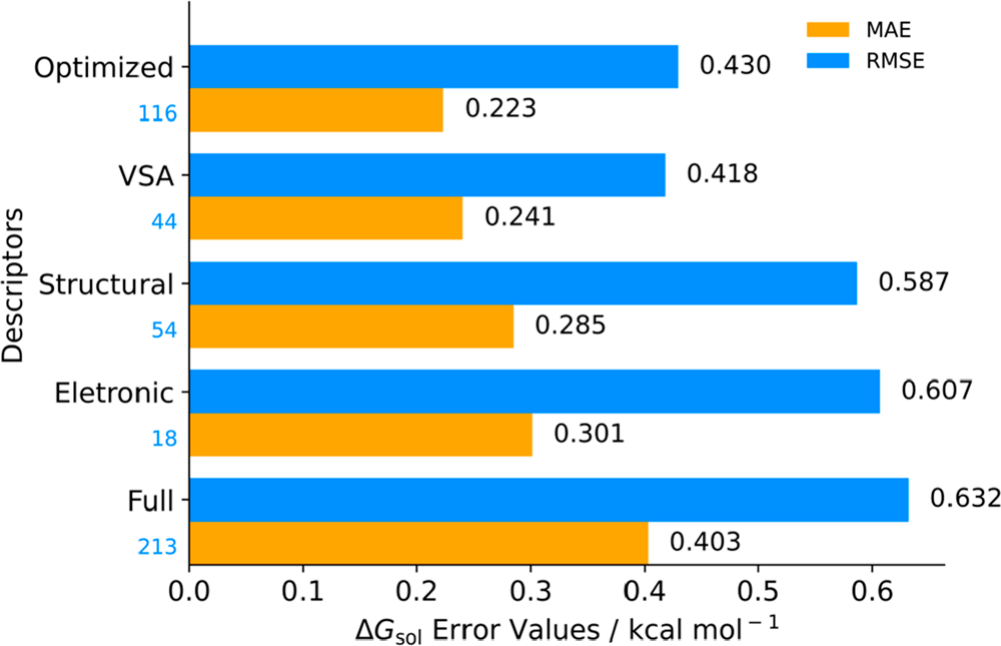
Δ*G*_sol_ prediction performance distribution across different descriptor groups: MAE, RMSE, and number of descriptors used (light blue). “Optimized” features represents the sum of VSA, Electronic, and Structural descriptors.

Given the extensive description on individual feature impact, we moved our analysis by selecting the best descriptor groups and their overall impact on free energy prediction. From the 213 descriptors overall, we selected the ones amassing positive permutation importance and sorted them into three groups (detailed in the SI). By removing offsetting descriptors, we kept the remaining features to perform property prediction. Then, through model refitting, we made new predictions with the same data sample used for model validation. Figure 9 presents the results for MAE and RMSE error calculation for all feature groups.

Figure 9 divides model performance for the GB algorithm with all descriptors (Full), with specific groups (VSA, Electronic, and Structural), and with optimized features. There is a noticeable improvement on prediction performance as feature selection is narrowed into groups, giving increasing performance to Electronic, Structural and VSA descriptors. More insightful is how these groups of only positive PI descriptors reduce prediction error, as MAE is roughly reduced by 50%. This statistical evidence adds further meaning toward the previously described descriptor performances. Within the initial data set, the model was able to grasp physical meaning behind feature calculations, enabling it to improve Δ*G*_sol_ predictions as these features were drawn from the starting database. Free energy predictions were improved by reducing the number of features, hence overall complexity, arguing against possible model overfitting. The different values for descriptor groups and the subsequent optimized value when they are integrated indicate that the model overlooked feature memorization and established predictions by feature correlations. This also enables an important feature selection for subsequent model optimization, improving from data set calculations.

Our findings materialize the notion of key characteristics of topological, descriptive, and electronic features, relevant toward Δ*G*_sol_ prediction. Our model has more difficulty assessing seldomly complex solute molecular structures, with low polarity and close to neutral electronic charge. Molecule size (χ and HeavyAtom descriptors predominantly) has associated higher predictions errors for the studied molecules. But perhaps we can name two important takeaways. First, the most important descriptors are not entirely codependent (aside from features with the same calculations methods), as prediction error stems from a wide combination of features, meaning that a sole descriptor cannot change the result. Second, our model can handle a diverse set of solvents, as it was impossible to rule out a particular group with higher incidence of calculation error. The outlined physical meaning of these findings, however, should be read with caution when considering other databases for model comparison. But their representativity seems to capture aspects of molecular shape and electronic features of each chemical entity.

### 3.3 Solvent Holdout Tests

**Figure 10:**
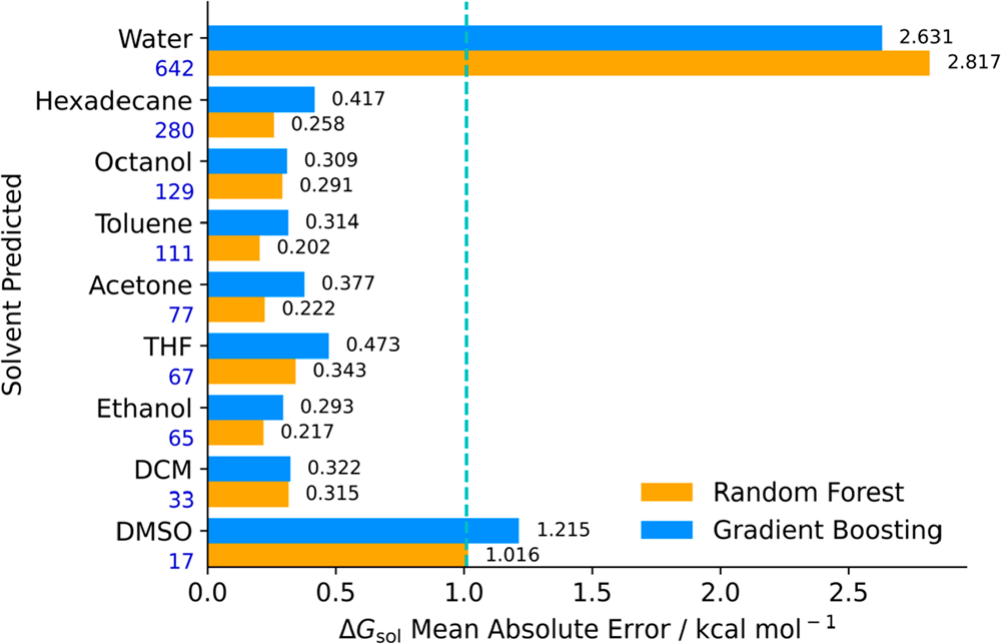
MAE for Δ*G*_sol_ prediction across different solvents in RF and GB algorithms, whose training set did not include the tested solvent. Blue line represents the 1 kcal mol^–1^ accuracy threshold.

To evaluate the wide scope of this model to unseen solvents, we present the results of a solvent holdout test by removing selected solvents from the model’s training set. The model calculation parameters were the same as selected from our optimization trials presented in previous sections and are described in the SI. The prediction MAE errors were determined as depicted in Figure 10. We compare our results against the 1 kcal mol^–1^ benchmark threshold for evaluation purposes.

There is a noteworthy contrast in Δ*G*_sol_ MAE between water and organic solvents. The presence of water as solvent greatly increases error prediction results relative to the benchmark baseline. For organic solvents, accurate results are evident in hexadecane, toluene, octanol, and other organic solvents of different polarities. Differences in calculated MAE and RMSE are almost negligible (and between algorithms), as all are below the 1 kcal mol^–1^ accuracy threshold. Considering the number of samples and wide distribution along the Δ*G*_sol_ scope of the database, it is possible to argue that without all of the training data the model is not able to grasp important explanatory power. An in-depth error inspection unveils that Δ*G*_sol_ prediction error over 1 kcal mol^–1^, with water as a solvent, displays a molecular refractivity between 5 and 50. The result overlaps with the previously explored MolMR values associated with higher prediction errors, as these values are removed from the training set when performing solvent holdout tests. This in turn leads to an increase in overall MAE error, remarkably different from other solvent holdout tests. However, these MAE results are very similar to other studies using the same ML methodology. Low et al.’s state-of-the-art GNN model yielded MAE Δ*G*_sol_ prediction errors around 2.12 kcal mol^–1^ with water as solvent, close to the 2.63 kcal mol^–1^ in our model. The GNN model presents mean absolute errors below 1 kcal mol^–1^ for organic solvents, just as depicted for our model (which outperforms Low et al.’s model for several solvents). With different distributions, both our and the GNN model might not cope with entropic contribution to solvation, given that solute polarizability could be a destabilizing factor in the solute–solvent pair, as it can influence entropic stabilization. The chemical behavior amassed in the entries with water as solvent highlights their relevance for being present in the training set, as the model was unable to learn this entropic contribution due to lack of representativity. To overcome this, more data on Δ*G*_sol_ entries with organic solvents across MolMR values in the 5–50 range should likely reduce prediction error.

These solvent holdout tests, along with the prediction performance distribution across different descriptor groups, can attest to our model’s resistance to overfitting. As the holdout error results compare favorably with GNN models and given the improved accuracy by reducing complexity by the number of features, we argue that our ML model is not prone to overfitting. With less feature information, the model was able generalize well on unseen data, not depreciating the overall accuracy result. Given our simple approach, the model can generalize important chemical information about solute–solvent interactions for similar solvents, yielding an encouraging motif toward the applicability of these descriptors.

### 3.4 Model Optimization

We proceeded with Model Optimization on both ensemble algorithms using only the most relevant features with PI > 0%, using the same train:test:validation split. Our results were recalculated accordingly, using optimized model algorithm parameters, and statistical results are presented in Table 2. The pipeline to determine the best algorithm parameters is presented in the SI.

**Table 2:** Results for Model Performance after Feature Optimization on Δ*G*_sol_ Prediction for RF and GB Algorithms

Regressor Algorithm	Score Train	Score Test	MAE/kcal mol^–1^	RMSE/kcal mol^–1^	SDP/kcal mol^–1^
Random Forest	0.95 ± 0.02	9.9 × 10^–5^ ± 1 × 10^–6^	0.26 ± 0.03	0.5 ± 0.1	0.11 ± 0.04
Gradient Boosting	0.964 ± 0.007	9.9 × 10^–5^ ± 9 × 10^–6^	0.22 ± 0.02	0.43 ± 0.07	0.09 ± 0.02

As expected, the overall prediction scores have improved. Both RF and GB algorithms had their train score revamped, with a best decrease in MAE prediction to 0.22 kcal mol^–1^, an RMSE of 0.43 kcal mol^–1^, and an SDP of 0.09 kcal mol^–1^. Using only roughly 50% of the starting descriptors, we maintained high feature correlation without compromising statistical accuracy.

Compared with state-of-the-art Neural Network Δ*G*_sol_ prediction models, Lim et al.’s model^5^ yielded a minimum prediction MAE of 0.19 kcal mol^–1^, while our model using a GB optimized algorithm presents a similar result. Our model accuracy also outperformed current DFT prediction methods with different solvation models, where a Neural Network benchmark model^61^ presented an absolute deviation error of 1.1 kcal mol^–1^.

**Figure 11:**
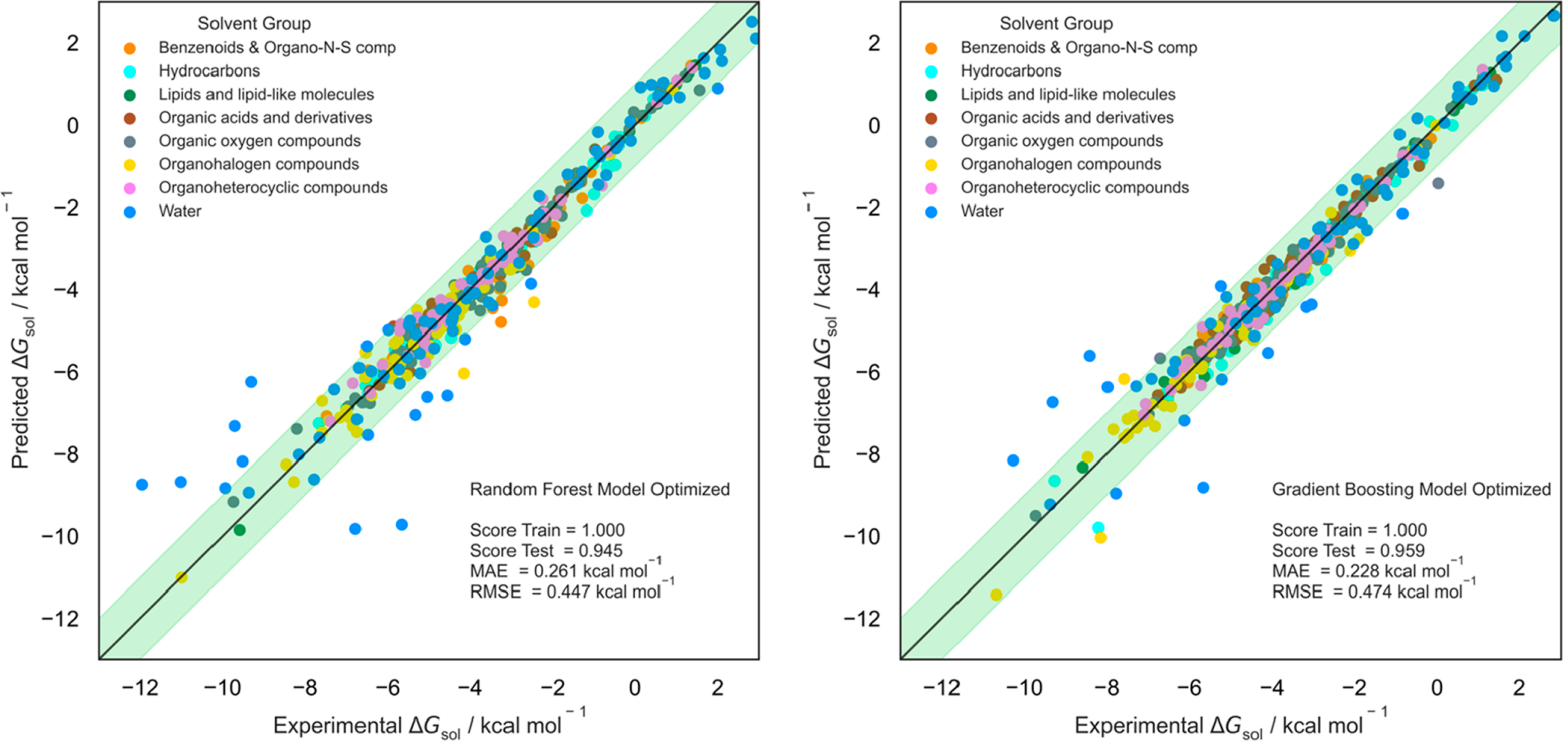
Fitness prediction plot for Δ*G*_sol_ prediction using an ML model with optimized RF (left) and GB (right) algorithms. Green band is within the 1 kcal mol^–1^ threshold.

Figure 11 depicts the graphical distribution of updated predictions that, compared with preoptimized results, showcases an improvement of test scores within the 1 kcal mol^–1^ threshold.

As expected, according to the solvent holdout tests, experimental points with water as solvent were harder to predict, especially on increasingly lower Δ*G*_sol_ values. This is also correlated with low data set points on these Δ*G*_sol_ prediction ranges, which also strengthens the overall prediction result.

### 3.5 Model Benchmarking

**Figure 12:**
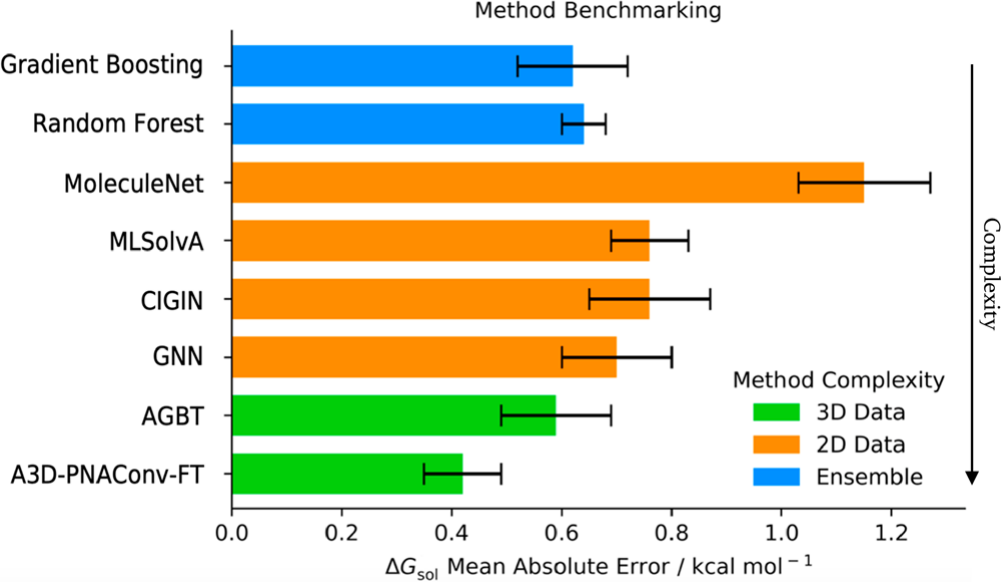
Model benchmarking with MAE for Δ*G*_sol_ prediction across different literature models. “Ensemble” represents this Article’s methods, while “3D” and “2D” denote models using 3D or 2D input data.

We compared our model with previously reported results using the FreeSolv data set with ML methods for benchmarking purposes. Despite having different levels of complexity, our models show similar prediction results using the same random 80:10:10 train:test:validation scheme. The details of method benchmarking are presented in the SI. Figure 12 depicts the prediction performance of our model with both ensemble algorithms against other methods with different method complexities.

We divided literature ML methods into two groups: (i) methods using 3D atomic features as input descriptors: A3D-PNAConv-FT^15^ model and AGBT;^62^ (ii) methods using GNN with 2D features from Low et al.^33^ (GNN), Pathak et al.^63^ (CIGIN), Lim et al.^5^ (MLSolvA), and Wu et al.^49^ (MoleculeNet). Both of our algorithms achieve an MAE close to 0.6 kcal mol^–1^, which outclasses most 2D data models. Only A3D-PNACONv-FT, using 3D data features, yields more favorable results with an MAE of 0.4 kcal mol^–1^. This is a significant difference, as the 3D models depicted here used thousands of additional DFT-calculated Δ*G*_sol_ values, as added structural information evidently improves model accuracy. But this comes with a trade-off on computational cost which sometimes is not compatible with the substantial increase of feature calculation. In comparison, it is interesting that without extensive additional information, our lower tier model could depict results to the same degree (despite the limited size of the original FreeSolv database). Compared with the original FreeSolv database average unsigned error of 1.14 ± 0.04 kcal mol^–1^, our lowest MAE of 0.60 kcal mol^–1^ ensures an improvement from initial database predictions. We thus argue that our model achieves a better trade-off between speed and accuracy, predicting reasonably accurate Δ*G*_sol_ predictions, without needing extensive input data.

As described in the previous sections, the added insight from feature description on the statistical predictions gives relevance to the explanatory power of our ML models. It is remarkable that these accurate results were achieved using simple input information from molecular structure and experimental measurements, enabling an ML model to learn specific intricacies of Δ*G*_sol_ prediction. This type of model using selected types of features using open-source software provides chemists a very powerful tool to predict a wide range of thermochemical properties, often difficult to experimentally determine. Without the need for specialized programming skillsets on model development and data set curation, we have presented a framework applicable for complex molecules using a variety of solvents. With an effective demonstration on describing key elements in chemical interpretation of theoretical predictions, these ML models can be further used in prediction of new molecules toward chemical feature testing. An interesting follow-up study should address how this model behaves for macromolecules, as this study only considered solvents and solutes up to 500 Da in open-source databases.

## 4 Conclusion

We have presented a data-driven ML model using regressor ensemble algorithms to accurately predict the Δ*G*_sol_ of an extensive solute–solvent database through explainable chemical feature insights. By using a wide range of structural, electronic, and surface area descriptors after sorting relevant contributors, model accuracy was improved for Δ*G*_sol_ prediction errors close to complex Neural Network ML models. Precision increases were made mainly on trimming outlier descriptors at both ends of Δ*G*_sol_ values, reducing overall best prediction MAE to 0.22 kcal mol^–1^ and RMSE to 0.43 kcal mol^–1^, well within comparable values of state-of-the-art models. However, our approach with supervised ensemble algorithms is user-friendly, simple, and faster, yielding precise predictions without extensive QM or ML additional calculations like most flagship Δ*G*_sol_ prediction models.

The separate chemical contributions grasped by our ML model through statistical performance of descriptors groups were shown to add important explanatory insights on assessing prediction accuracy. Solute electronic and surface area interactions provided key information on prediction outcome relative to base models without optimization, adding experimental awareness on molecular behavior. This explanatory information concerning increasing Δ*G*_sol_ predictions will guide chemists with important evidence in experimental design and add further credibility to computational predictions on complex thermodynamic properties. The additional validation assays on solvent holdout tests and database benchmarking further densify the model’s explanatory remarks, correlating Δ*G*_sol_ predictions with the solvent’s particular characteristics and solute structural descriptions. Although the model does not grasp the full plenitude of experimental Δ*G*_sol_ results, further prediction improvements can be achieved by tallying additional data on top of this initial data set, mainly on organic solvents and solutes with increasing polar surface area and decreasing polarizability.

This ML-based strategy constitutes a relevant development in AI modeling for complex thermodynamic property predictions. With this step forward, we have managed to balance speed and accuracy in Δ*G*_sol_ predictions by using available, open-source, and transferable input data, producing explanatory calculations which can be broadly expanded toward chemical modeling and property prediction.

## Supporting Information Available

List of compounds used in the model’s database, algorithm, and model optimization Features, RDKit Full Descriptor List, and the repository for the sample code for model design. (PDF); Complete list of statistical results: model calculations, feature importances, solvent holdout tests and descriptor importance ranking (XLSX)
