# Supplementary material for: Explainable Supervised Machine Learning Model To Predict Solvation Gibbs Energy
Source: J Chem Inf Model. 2023 Aug 21;64(7):2250–62. doi: 10.1021/acs.jcim.3c00544 (PMC11005042; doi:10.1021/acs.jcim.3c00544)
Supplement: Supplementary file 1 — ci3c00544_si_001.pdf [file ci3c00544_si_001.pdf]

SUPPORTING INFORMATION

Explainable Supervised Machine Learning Model to Predict Solvation Gibbs Energy

José Ferraz-Caetano[1], Filipe Teixeira[2], M. Natália D. S. Cordeiro[1]

1) LAQV-REQUIMTE – Department of Chemistry and Biochemistry – Faculty of Sciences, University of Porto - Rua do Campo Alegre, S/N, 4169-007 Porto, Portugal

2) CQUM – Centre of Chemistry, University of Minho, Campus de Gualtar, 4710-057 Braga, Portugal

*Corresponding Authors: **José Ferraz-Caetano (**jose.caetano@fc.up.pt); **M. Natália D. S. Cordeiro[2]** (ncordeir@fc.up.pt)

INDEX

Annex S1 – Model and External Database S2
Annex S2 – Algorithm and Model Optimization Features S2
Annex S3 – RDKit Full Descriptor List S3
Annex S4 – Sample Code for ML Model Design S5
REFERENCES S6

Annex S1 – Model and External Database

Table A1. List of compounds used in the model's database.

A database of all solvent:solute pairs is available at the zenodo repository[1]: https://doi.org/10.5281/zenodo.8121619

Annex S2 – Algorithm and Model Optimization Features

Best hyper-parameters for each algorithm for model optimization:

Random Forest Regressor = n_estimators= 250, min_samples_split= 2, min_samples_leaf= 1, max_features='sqrt', max_depth= None, bootstrap= False, random_state=47

Gradient Boosting Regressor = n_estimators= 250, min_samples_split= 5, min_samples_leaf= 5, max_features= 'sqrt', max_depth= None, random_state=47

Model Benchmarking procedure:

Using the same initial FreeSolv dataset[2], we trained our presented ML model with the FreeSolv data, using the same random train-test-validation split of 80:10:10, suggested by MoleculeNet authors[3] and replicated in the other quoted methods.

Annex S3 – RDKit Full Descriptor List

Descriptors used in Machine Learning model development (Table S1). The respective bibliographical foundation of each descripted is described in detail in the RDKit WebBook Documentation[4]. The descriptor calculations were made by converting each SMILES string (representing each molecular entry), running on top of Python version 3.9[5] and using RDKit package, version 2022.09.4[6].

Table S1

List of Descriptors Used in Machine Learning model development

Descriptor	Type	Meaning
**BalabanJ**	Structural	Distance sum of the two end-vertex for each edge. BalabanJ index has been proven to be relevant to network branching.
**BertzCT**	Structural	Complexity index, taking into account both the variety of kinds of bond connectivities and atom types; information contents related to bond connectivity and atom type diversity.
**0χ , 1χ**	Structural	This descriptor signifies a retention index (zero order) derived directly from gradient retention times.
**0χn – 4χn**	Structural	This descriptor signifies a retention index (zero order) derived directly from gradient retention times.
**0χv – 4χv**	Structural	This descriptor signifies atomic valence connectivity index (order 0).
**VSA_EState1 – VSA_EState11**	Surface Area	MOE-type (QSAR model) descriptors using EState indices and surface area contributions.
**Hall Kier α**	Structural	Descriptor fingerprint that displays the difference between active and inactive molecules.
**HeavyAtomCount**	Structural	The number of heavy atoms in the molecule.

**HeavyAtomMolWt**	Structural	The average molecular weight of the molecule ignoring hydrogens.
κ_1 , κ_2 , κ_3	Structural	This descriptor signifies # κ shape index: (n-1) × 2 / m^2
Max and Min AbsEStateIndex	Electronic	Maximum and minimum Absolute E-State
Max and Min AbsPartialCharge	Electronic	Maximum and minimum Absolute Partial Charge
Max and Min EStateIndex	Electronic	Maximum and minimum E-State
Max and Min PartialCharge	Electronic	Maximum and minimum Partial Charge
**Mol logP**	Structural	Wildman-Crippen logP value.
**MolMR**	Electronic	Wildman-Crippen molar refractivity.
**MolWt**	Structural	The average molecular weight of the molecule.
**NHOH Count**	Structural	The number of NHs or OHs.
**NO Count**	Structural	The number of Nitrogens and Oxygens.
$n_{alicarb}$	Structural	The number of aliphatic carbocycles.
n_{alihet}	Structural	The number of aliphatic heterocycles.
n_{alirig}	Structural	The number of aliphatic rings.
$n_{arocarb}$	Structural	The number of aromatic carbocycles.
n_{arohet}	Structural	The number of aromatic heterocycles.
n_{arorig}	Structural	The number of aromatic rings.
n_{Ha}	Structural	The number of Hydrogen Bond Acceptors.
n_{Hd}	Structural	The number of Hydrogen Bond Donors.
n_{het}	Structural	The number of Heteroatoms.
n_{radele}	Structural	The number of radical electrons.
n_{rot}	Structural	The number of Rotatable Bonds.

n_{satrig}	Structural	The number of saturated rings.
n_{ele}	Structural	The number of valence electrons.
**PEOE_VSA1 – PEOE_VSA14**	Surface Area	MOE-type (QSAR model) descriptors using partial charges and surface area contributions.
**SMR_VSA1 – SMR_VSA10**	Surface Area	MOE-type (QSAR model) descriptors using MR contributions and surface area contributions.
**SlogP_VSA1 – SlogP_VSA12**	Surface Area	MOE-type (QSAR model) descriptors using logP contributions and surface area contributions.
**TPSA**	Surface Area	The total polar surface area of a molecule based upon fragment calculations.
**VSA_EState1 – VSA_EState10**	Surface Area	MOE-type (QSAR model) descriptors using EState indices and surface area contributions.

Annex S4 – Sample Code for ML Model Design

Table S2

List of links for the Database and Model development.

Model	Website
**Gibbs Free Energy Database**	https://github.com/jfcaetano/GibbsML
**Model Development**	https://github.com/jfcaetano/GibbsML

REFERENCES

1. Ferraz-Caetano, J.; Teixeira, F.; Cordeiro, M. N. D. S. ΔG-RDKit: Solvation Free Energy Database. http://dx.doi.org/10.5281/ZENODO.8121619 (accessed 10 July 2023).

2. Mobley, D. L.; Guthrie, J. P., FreeSolv: a database of experimental and calculated hydration free energies, with input files. *Journal of Computer-Aided Molecular Design* **2014,** *28* (7), 711-720.

3. Wu, Z.; Ramsundar, B.; Feinberg, Evan N.; Gomes, J.; Geniesse, C.; Pappu, A. S.; Leswing, K.; Pande, V., MoleculeNet: a benchmark for molecular machine learning. *Chemical Science* **2018,** *9* (2), 513-530.

4. RDKit: Open-source cheminformatics - Descritptor Webbook. https://www.rdkit.org/docs/GettingStartedInPython.html#list-of-available-descriptors (accessed March 1, 2022).

5. Python Software Foundation - Python Language Reference, version 3.9.8. http://www.python.org.

6. Landrum, G. RDKit: Open-source cheminformatics 2022_09_4 (Q3 2022) Release - January 16, 2023. http://www.rdkit.org/ (accessed January 18, 2023).
